# Tumor necrosis factor receptor-associated cycle syndrome: a case report and literature review

**DOI:** 10.3389/fped.2023.1296487

**Published:** 2023-12-13

**Authors:** Ziwei Li, Xiaoping Jing, Shuya Zhang, Tiantian Liu, Qingyin Guo

**Affiliations:** ^1^ The First Clinical Medical College, Henan University of Chinese Medicine, Zhengzhou, China; ^2^ Department of Traditional Chinese Medicine, School of Medicine, Shanghai Children’s Hospital, Shanghai Jiao Tong University, Shanghai, China; ^3^ Department of Pediatrics, The First Affiliated Hospital of Henan University of Chinese Medicine, Zhengzhou, China

**Keywords:** TNF, heredity, syndrome, periodic fever, children, literature review, case report

## Abstract

Recurring episodes of fever characterize tumor necrosis factor receptor-associated periodic syndrome (TRAPS) which is autosomal dominant. The primary symptoms of patients with TRAPS include prolonged fever, abdominal pain, muscle pain, and skin rashes. The prevalence of TRAPS appeared higher in Western countries than in Asian countries. Herein, we present the case of a 13-year-old girl who experienced intermittent fever for 8 years, with episodes that occur every 2 years. The patient demonstrated periodic fever, headache, vomiting, rash, and elevated inflammatory marker levels during the disease course. A heterozygous C55Y mutation was identified via a direct DNA sequencing of her genomic DNA. This mutation is located in exon 4 of TNFRSF1A. Genetic studies of her sister and mother revealed that they possessed the C55Y heterozygous mutation without demonstrating any clinical signs, while the father did not. Further, we conducted a thorough assessment of the literature and compiled the information from the eight TRAPS case series.

## 1. Introduction

TRAPS is an autosomal dominant autoimmune syndrome associated with a heterozygous TNFRSF 1A gene variant, which encodes the tumor necrosis factor receptor 1 (TNFR 1) receptor (1). TRAPS is characterized by periodic fever, sometimes lasting for several weeks, accompanied by long-term or recurrent abdominal pain, muscle pain, joint pain, migratory erythematous rash, eye inflammation, lymph node enlargement, and headache episodes (2). Physical stress or emotions, mild infection, fatigue, hormonal changes, trauma, or vaccination may trigger these symptoms, but the cause remains unknown. Studies that include Asians are very rare, with very few reports in China. This case report aims to determine the clinical characteristics, diagnostic process, treatment strategies, and review relevant literature of TRAPS.

## 2. Case report

A 13-year-8-month-old girl had intermittent fever, with a 5-day recurrence, for >8 years. She was presented to our pediatric department for retreatment on May 9, 2023. Five days earlier, she experienced nausea and vomiting with an unknown cause and with no accompanying symptoms, such as fever, dizziness, headache, or blurred vision. Four days earlier, she had a fever with a peak temperature (*T*-max) of 39°C, but the fever intermittently continued. Physical examination revealed dark red ecchymosis on the abdomen, mild headache in the frontal and occipital regions, and swollen tonsils to grade III. Biochemical examination revealed a white blood cell (WBC) count of 12.3 × 10^9/L^, a C-reactive protein (CRP) level of 69.7 mg/L, and a strongly positive rheumatoid factor immunoglobulin M (IgM) of 104.97 RU/ml. All blood tests during hospitalization are shown on the Supplementary Table S1. Ultrasonography revealed gallbladder polyps, a solid thyroid nodule (TI-RADS class 3), and cysts in both thyroid lobes. Computed tomography (CT) revealed bone marrow edema below the sacral bone on the right side. Chest x-ray, urine routine, blood routine, cerebrospinal fluid test, T, B, and natural killer lymphocyte subsets, and bone marrow aspiration revealed normal results.

In June 2015, the patient had a fever with an unknown cause and was hospitalized for 2 weeks, with a T-max of 40.6°C and with no rashes or headaches. Blood routine examination revealed WBC of 19.47 × 10^9/L^, neutrophils (*N*) of 82.5%, CRP level of 107 mg/L, and erythrocyte sedimentation rate (ESR) of 64 mm/h. She was then diagnosed with sepsis and myocardial injury. After >20 days of antibiotic treatment, her temperature and inflammation indicators returned to normal, and she was discharged. In September 2015, she had a fever with scattered red papules around the umbilicus and was hospitalized. *T*-max is 38.6°C, WBC is 12.33 × 10^9/L^, *N* is 69%, CRP level is 24 mg/L, and ESR is 21 mm/h. She was diagnosed with “sepsis.” After 13 days of antibiotic treatment, her temperature and inflammation indicators returned to normal levels, and she was discharged. In June 2017, she had a fever with vomiting. *T*-max is 40.1°C, WBC is 16.7 × 10^9/L^, *N* is 85.5%, CRP level is 117 mg/L, and ESR is 35 mm/h. She was diagnosed with “septicemia.” After >20 days of antibiotic treatment, her temperature and inflammation indicators returned to normal, and she was discharged. In February 2021, she had a fever with an unknown cause and was hospitalized. *T*-max is 40°C, WBC is 29.7 × 10^9/L^, *N* is 95.6%, and CRP level is 249 mg/L (Table 1). The rheumatoid factor, IgM, was 207.13 RU/ml (strongly positive). The patient's inflammation indicators and body temperature returned to normal levels after >20 days of antibiotic therapy, and she was discharged.

**Table 1:** Blood test records of the patient at the time of onset.

	WBC (10^9/L^)	*N*%	CRP (mg/L)	ESR (mm/h)
2015-06	19.47	82.5	107	64
2015-09	12.33	69	24	21
2017-06	16.7	85.5	117	35
2021-02	29.7	95.6	249	66
2023-05	12.1	70.7	69.3	64

**Figure 1:**
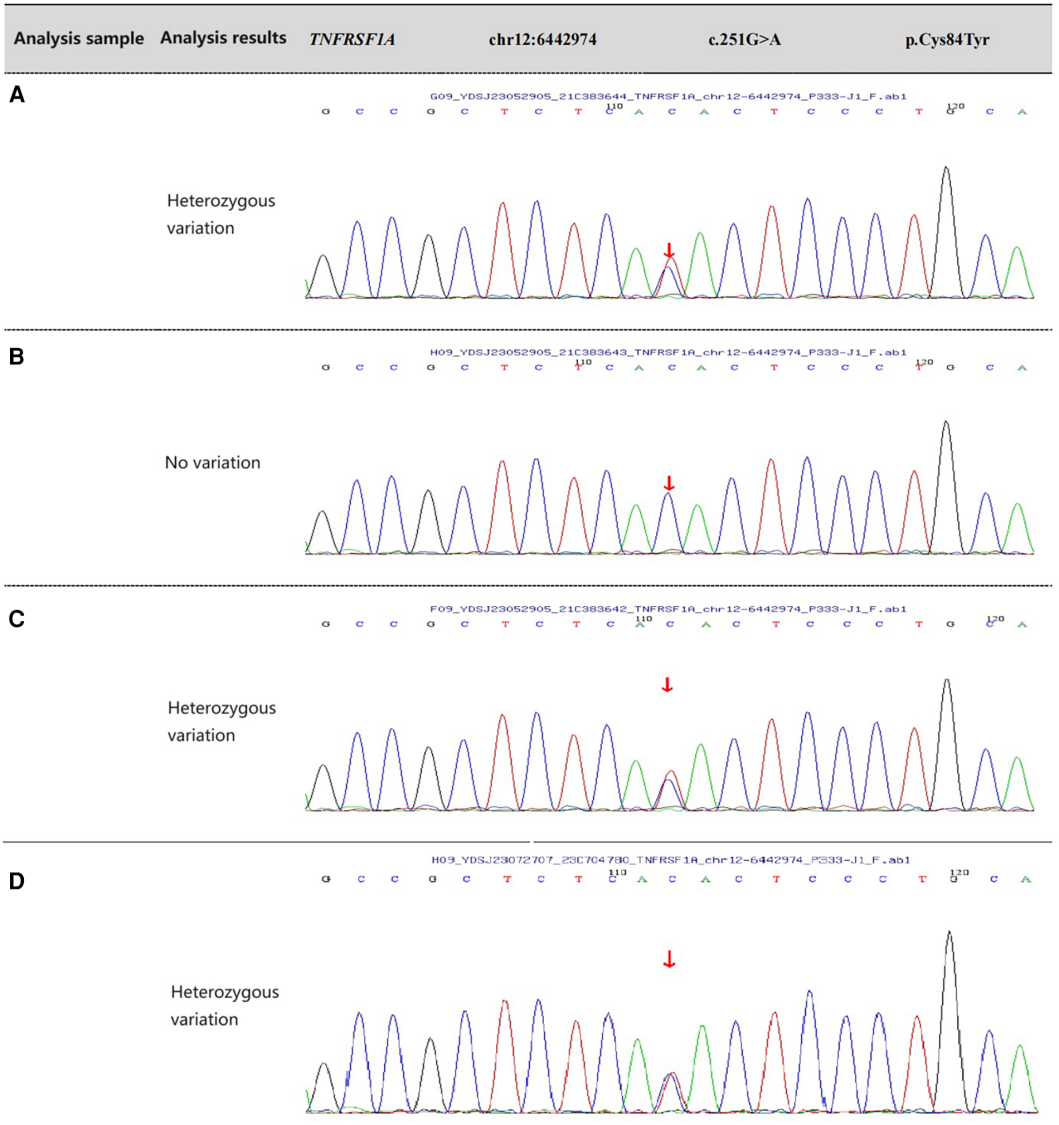
Gene sequencing results of the patient and her relatives. (**A**) The patient has a mutation in the TNFRSF1A gene, with nucleotide G at position 251 replaced by A, resulting in the C55Y substitution. (**B**) The father of the patient has no variation in the TNFRSF1A gene. (**C**) The mother of the patient has a mutation in the TNFRSF1A gene. (**D**) The sister of the patient has a mutation in the TNFRSF1A gene. The arrow indicates the mutation site.

Because of the clinical manifestations and symptoms of the patient, showing periodic fever, headache and skin ecchymosis and other symptoms, the inflammatory reactants increased during the acute phase of the attack and normal during the asymptomatic period. There were no abnormalities in DNA pathogenic microorganism detection, urine routine, blood biochemistry, TBNK lymphocyte subsets, thoracic orthostatic DR, bone penetration and cerebrospinal fluid culture. Overall consideration was systemic inflammatory response syndrome. The hope is that genetic studies will confirm more accurate diagnoses and provide targeted treatment recommendations. Sanger sequencing was performed on the patient and his father, mother and sister for verification. Through sequencing analysis of disease-related genes, highly suspicious variants associated with the disease phenotype were identified. The results of the nuclear gene analysis revealed a heterozygous mutation in the TNFRSF1A gene in the patient. Specifically, a heterozygous mutation at nucleotide 251, where G was changed to A (c.251G> A), resulting in the substitution of cysteine with tyrosine at amino acid position 84 (p.Cys84Tyr), also known as Cys55Tyr or C55Y. The heterozygous variant was found at the same location in the patient's mother and sister, while no variant was found at that location in the father (Figure 1).

Treatment process: Based on the patient's medical history and examination, she was admitted to the hospital and received treatment with ibuprofen in combination with cefoperazone sodium and sulfate sodium for anti-infection therapy. However, her fever did not subside. After 7 days, she was started on oral prednisone acetate tablets (15 mg three times a day). One day later, her body temperature returned to normal, and two days later, the dosage of prednisone acetate tablets was reduced to 3 mg three times a day. The patient was followed up during the six-month period after discharge, and there was no recurrence.

## 3. Discussion

The clinical characteristics in our instance, which include recurrent fever bouts lasting at least one week, rash, and headache symptoms, are compatible with TRAPS.WBC and CRP increase, while ESR rapidly increases, during the acute phase. After treatment with prednisone acetate tablets, the body temperature returned to normal. Genetic testing revealed a heterozygous C55Y mutation in exon 4 of the TNFRSF1A gene in the patient. Previous publications have connected this mutation to TRAPS, and its genetic pathogenicity has been proven (3). This patient therefore meets the diagnostic criteria for TRAPS (4). To our knowledge, this is the first case of C55Y mutation in Asia.

**Figure 2:**
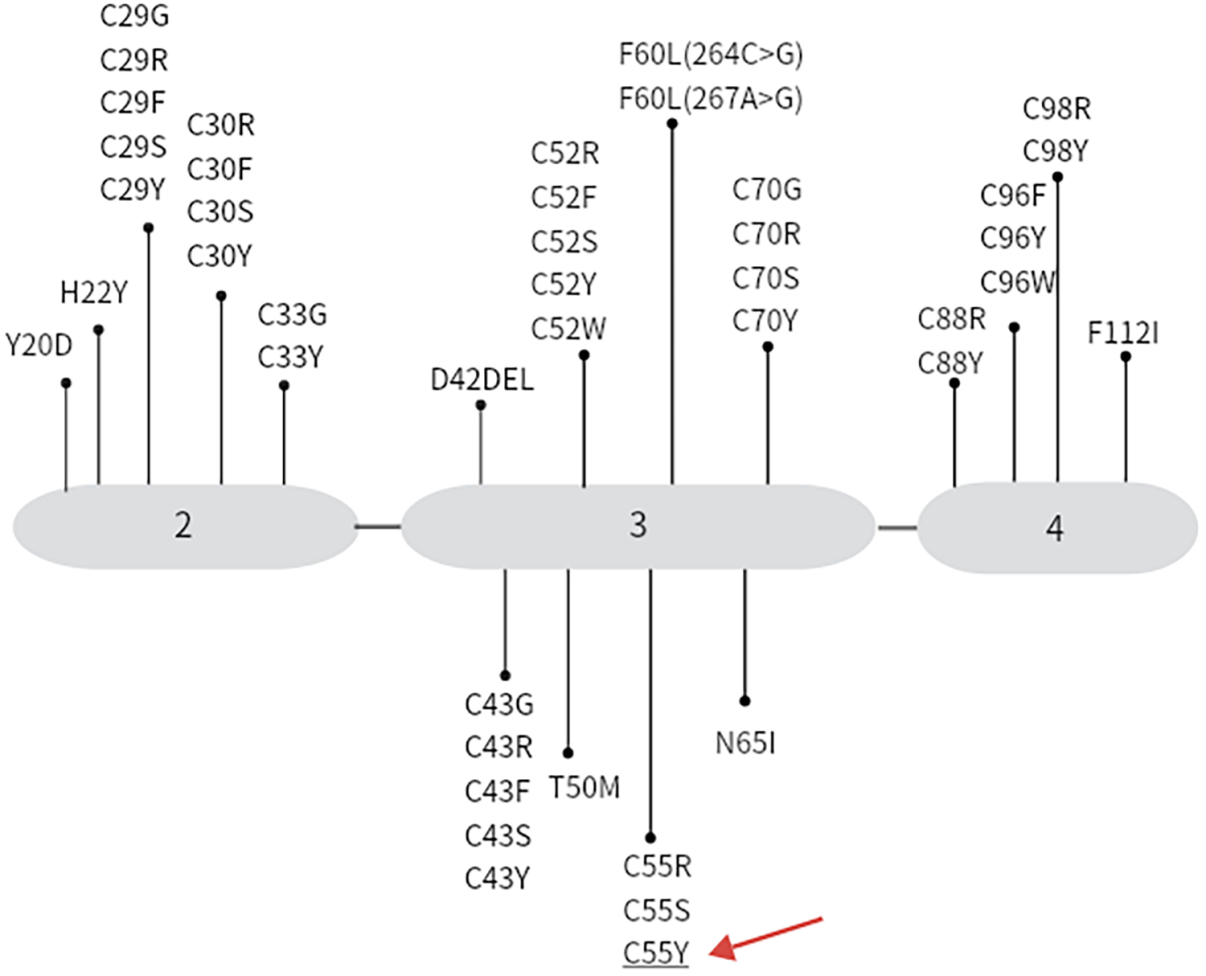
Shows the 44 missense variants that have been confirmed as pathogenic in the infevers database. The gene mutation type of the patient in this case is indicated by the red arrow.

The first instance of TRAPS, which was first referred to as “familial periodic fever,” occurred in an Irish-Scottish family in 1982. Its clinical phenotype is very similar to familial Mediterranean fever (FMF) (5). The term was renamed to TRAPS in 1999 following the identification of disorders brought on by spontaneous mutations in the TNFR gene. The TNFRSF1A gene has 10 exons and is found on chromosome 12p13. This autosomal dominant disorder is brought on by a missense mutation in exon 10 of the TNFRSF1A gene, which codes for the 55 kDa TNFR (6). So far the Infevers database (Infevers TRAPS database http://fmf.igh.cnrs.fr/infevers) is described in the 183 TNFRSF1A missense mutation of genes. Among these, 62 variations have been identified as possibly pathogenic (7), and 44 missense variants have been confirmed as pathogenic (Figure 2). The pathogenesis of TRAPS is highly complex, involving the assembly of signaling pathways upon binding of TNFα to TNFR1, ultimately upregulating the expression of many pro-inflammatory cytokine genes. Heterozygous variants affect the structure of the extracellular domain and its ability to bind the TNF ligand. Unfolded protein response (UPR), endoplasmic reticulum stress, and mitochondrial reactive oxygen species (ROS) generation are all upregulated as a result of the misfolded protein that results from the mutation accumulating in the cell (8). If the stress is too severe or lasts too long, it may lead to cellular damage and inflammatory responses.

**Figure 3:**
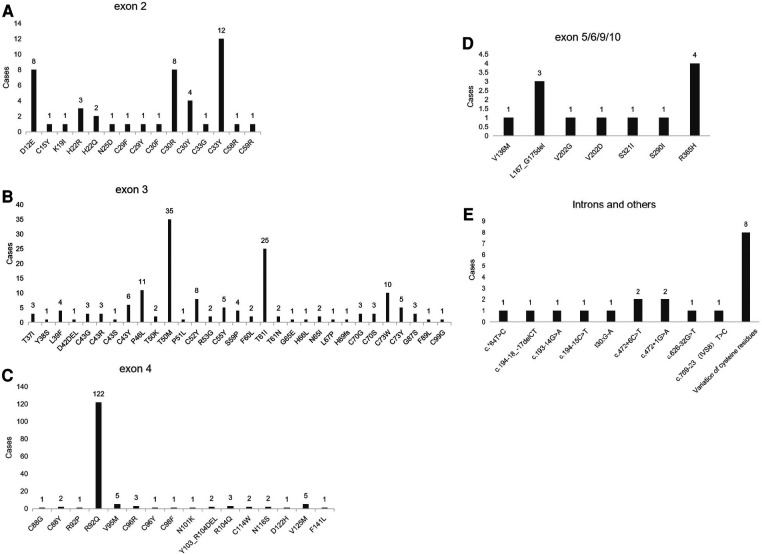
Shows the genotypes of TNFRSF1A variants in patients. (**A**) Represents the genotype with TNFRSF1A variants in exon 2; (**B**) represents the genotype with TNFRSF1A variants in exon 3; (**C**) represents the genotype with TNFRSF1A variants in exon 4; (**D**) represents the genotype with TNFRSF1A variants in exons 5, 6, 9, and 10; (**E**) represents the genotype with TNFRSF1A variants in introns and other positions.

The search was conducted in PubMed database with the keyword “Tumor necrosis factor receptor-associated periodic syndrome” from January 1, 2010 to August 15, 2023. A total of 104 literatures were retrieved, and 53 were included after eliminating duplicate reports and insufficient data. Eight studies (more than five cases) totaling more than five cases were finally considered for cohort analysis (9–16), which provided comprehensive clinical descriptions of the variations harbored by the patients. We reviewed patients from the following cohorts: a total of 381 patients were analyzed, with 17 clinical features. The most common clinical manifestations included fever (84%), abdominal pain (60%), arthralgia (52%), myalgia (50%), erythematous rash (47%), arthritis (46%), chest pain (32%), acute conjunctivitis (20%), headache (21%), sore throat (18%), lymph nodes enlargement (15%), periorbital oedema (14%), pericarditis (8%), oral aphthosis (8%), amyloidosis (8%), and hepatosplenomegaly (3%) (Table 2). The R92Q variant, which is found in 122 cases, is the most prevalent mutation type among these patients according to this article's summary. The majority of gene mutations are located in exon 3 (Figure 3). Gaggiano et al. (9) analyzed the literature of 80 patients with TRAPS from around the world and found that the children group reported a positive family history of recurrent fever more frequently than the adult group. Regarding the clinical features during an attack, pericarditis and myalgia are more common in adults, while abdominal pain is more common in children. Moreover, high visibility (HP) mutational symptoms during active disease are always strongly associated with increases in acute reactants (ESR, CRP, serum amyloid-A, fibrinogen, and ferritin (17). This article also shows that the clinical presentation of the syndrome is influenced more by the penetrance of the mutation than by the age of onset itself. Cantarini et al. (10) reported that individuals with low-penetrance TNFRSF1A variants had a lower family history of inflammatory attacks compared to patients with structural mutations, and they exhibited a later onset of disease, but there was no difference in this regard between individuals with and without a genetic diagnosis. Zhao et al. (11) reported the first and largest case series of TRAPS in Chinese adult patients. It was observed that there is a significant difference in the gender ratio between male and female patients with this disease (7:2), which differs from previous research findings (1:1). The study also suggests that the variations in clinical manifestations among TRAPS patients in different regions may be related to differences in TNFRSF1A genotypes. Another common symptom of this disease is recurrent rashes, which are more commonly observed in Chinese patients compared to patients from Japan and Europe. In a study conducted by Ueda et al. (12) in Japan, it was found that fever, joint pain, and rash are more commonly observed symptoms in patients with TNFRSF1A variations. In comparison to Caucasian patients, they have a lower prevalence of abdominal pain, amyloidosis, and muscle pain. The prevalence of abdominal pain, myalgia, and amyloidosis was significantly lower in Japanese patients compared to Caucasian patients. The T61I variant was the most common. Nezos et al. (13) conducted the first lineage analysis of TRAPS in Greece and identified a rare C73Y mutation. More severe symptoms are associated with the TNFRSF1A disulfide bond. C73Y, C73W and T50M have more severe clinical manifestations. Lachmann et al. (14) reported the largest case series study to date, the variant is R92Q in 34% of patients. The most common features are fever, myalgia, rash, abdominal pain, and ocular manifestations. Patients with the R92Q variant had a late onset. A survey by Jesus et al. (15) indicated a median fever duration of 5 days in TRAPS.

**Table 2:** Bed characteristics of patients with receptor-associated periodic syndrome.

Demographic characteristics and clinical manifestations	Gaggiano (11) (*n* = 80)	Cantarini (12) (*n* = 49)	Zhao (13) (*n* = 9)	Ueda (14) (*n* = 51)	Nezos (15) (*n* = 22)	Lachmann (16) (*n* = 158)	Jesus (17) (*n* = 7)	Vergara (18) (*n* = 5)
Gender (Male/female)	42:38	25:24	7:2	22:29	–	78:80	6:1	2:3
Onset age	3 (0.5–38.5)	20.25 ± 16.15	9	–	–	4.3 (0–63)	4.8 (0.3–12)	(0.25–21)
Fever	59 (74%)	47 (96%)	9 (100%)	44 (100%)	15 (68%)	133 (84%)	7 (100%)	5 (100%)
Abdominal pain	56 (70%)	21 (43%)	5 (55.6%)	16 (36%)	13 (59%)	111 (70%)	5 (71%)	3 (60%)
Arthralgia	10 (12.5%)	32 (65%)	5 (55.6%)	26 (59%)	15 (68%)	101 (64%)	6 (86%)	3 (60%)
Myalgia	8 (10%)	32 (65%)	6 (66.7%)	19 (43%)	6 (27%)	111 (70%)	4 (57%)	4 (80%)
Erythematous rash	33 (41%)	9 (18%)	7 (77.8%)	24 (55%)	3 (13.6%)	96 (63%)	4 (57%)	3 (60%)
Arthritis	18 (22.5%)	5 (10%)	5 (55.6%)	26 (59%)	13 (59%)	101 (64%)	4 (57%)	3 (60%)
Acute conjunctivitis	14 (12.5%)	14 (29%)	4 (44.4%)	8 (18%)	1 (4%)	35 (22%)	–	2 (40%)
Periorbital oedema	14 (12.5%)	–	2 (22.2%)	4 (9%)	–	32 (20%)	3 (43%)	–
Sore throat	24 (30%)	6 (12%)	6 (66.7%)	–	–	33 (21%)	–	–
Lymph nodes enlargement	30 (38%)	10 (20%)	2 (22.2%)	–	–	13 (8%)	–	2 (40%)
Hepatosplenomegaly	9 (11%)	–	–	–	–	–	2 (28.5%)	–
Chest pain	53 (66%)	20 (41%)	0	6 (13.6%)	–	40 (25%)	2 (28.5%)	–
Headache	8 (10%)	20 (41%)	5 (55.6%)	10 (23%)	–	36 (23%)	–	–
Pericarditis	18 (22.5%)	13 (27%)	–	–	–	–	–	–
Oral aphthosis	20 (25%)	9 (18%)	–	–	–	–	–	–
Amyloidosis	8 (10%)	6 (12%)	–	0	1 (4%)	16 (10%)	–	–
Vomiting	2 (2.5%)	–	–	–	–	–	–	–

In 2019, a new classification criteria for TRAPS was reported (4). The new criteria for TRAPS include: the presence of a confirmed TNFRSF1A genotype and at least one of the following features: attacks lasting more than 7 days/myalgia/migratory rash/periorbital edema/family history. In the absence of a confirmed TNFRSF1A genotype, at least two of the following clinical characteristics must be met: attacks lasting more than 7 days/myalgia/mobile rash/periorbital edema/family history. The combination has a sensitivity of 95%, specificity of 99%, and accuracy of 99%. Due to the rarity of this disease, diagnosis is often delayed, and to this day, there is only one case series analysis of TRAPS in China. The child described in this article was not diagnosed with TRAPS until the 8th year of onset, when genetic testing was finally performed. This reminds clinicians to strengthen the re-understanding of the disease, improve the level of diagnosis and treatment, in order to reduce the occurrence of misdiagnosis.

The treatment goal for TRAPS is to control symptoms and prevent long-term complications. According to a study (18), high doses of NSAID have some effect on the symptoms of some patients, while immunomodulators (cyclosporine, methotrexate) or colchicine have little effect (18). One group of medications utilized in the treatment of TRAPS is TNF-α inhibitors, such as etanercept. These medications have shown favorable safety profiles and demonstrated efficacy (19). Another category of drugs includes long-acting agents that target interleukin-1 (IL-1), such as Anakinra and Canakinumab (20). By selectively blocking immune mediators, these drugs effectively suppress disease activity, prevent amyloidosis, and halt organ damage (2). Since IL-6 levels may be elevated in trap (21), it has been hypothesized that monoclonal antibodies against the interleukin-6 (IL-6) receptor, may be relieve clinical symptoms (22).

How do we diagnose it clinically? The first is to know the family history of the patient. TRAPS usually run in families, and if there are multiple members of the family who have recurrent fever, myalgia, joint pain, rash, and other related symptoms, the likelihood of suspecting TRAPS is high. Second, understand the symptoms. People with TRAPS often have fever episodes that begin in childhood or adolescence and often last for a long time 1–3 weeks. Symptoms such as joint pain, rash, and lymph node enlargement are associated during the attack, and the symptoms can be alleviated by themselves after the attack. It was also associated with elevated WBC, CRP and ESR. These typical symptoms and test indicators should also arouse the suspicion of clinicians. The third point is to rule out other diseases: the symptoms of TRAPS are similar to those caused by many other autoimmune diseases and infections. Clinicians rule out other possibilities, such as rheumatoid arthritis, infectious diseases, etc., before considering the TRAPS gene test. If a clinician suspects a patient may have TRAPS, genetic testing can provide a definitive diagnosis to help guide treatment and management. Early diagnosis, standardized treatment, and effective management of symptoms and complications can enhance the quality of life for individuals affected by TRAPS, while also alleviating the economic burden on both society and families.

## Data Availability

The datasets presented in this study can be found in online repositories. The names of the repository/repositories and accession number(s) can be found below: https://www.ncbi.nlm.nih.gov/clinvar/variation/97673/. GenBank VCV000097673.13.
