# Supplementary material for: Tumor necrosis factor receptor-associated cycle syndrome: a case report and literature review
Source: Front Pediatr. 2023 Dec 13;11:1296487. doi: 10.3389/fped.2023.1296487 (PMC10753791; doi:10.3389/fped.2023.1296487)
Supplement: Supplementary file 1 [file Table1.pdf]

Supplementary Table 1

	WBC(10^9/L)	NEU%	LYM%	CRP(mg/L)	MCV(fL)	MCH(pg)	MCHC(g/L)	PCT(%)
2023-05-08	12.3	65.3	27.4	69.7	75.2	22.6	301	0.324
2023-05-10	13.4	63.6	30.4	74.3	73.2	22.4	305	0.350
2023-05-13	14.8	73.9	20.5	73.5	73.0	23.0	315	0.313
2023-05-15	14.6	79.0	17.7	78.4	71.2	22.6	318	0.346
2023-05-16	15.4	75.8	20.9	82.4	73.2	22.6	309	0.357
2023-05-17	21.9	85.5	12.5	60.9	74.6	23.1	310	0.410
2023-05-18	22.7	89.4	8.1	69.3	74.4	22.6	304	0.491
2023-05-19	28.2	88.5	8.8	29.9	76.9	23.0	299	0.516
